# The implications of measuring lipoprotein(a) in clinical practice

**DOI:** 10.21542/gcsp.2024.40

**Published:** 2024-08-01

**Authors:** Mahmoud Barbir, Alison Pottle, Stefan R. Bornstein

**Affiliations:** 1 Harefield Hospital, part of Guy’s and St Thomas’ NHS Foundation Trust, UK; 2 University Hospital Carl Gustav Carus Dresden, Fetscherstraße 74, 01307 Dresden, Germany; 3 King’s College London, Strand London WC2R 2LS

## Abstract

Lipoprotein(a) (Lp(a)) is a well-recognized causal risk factor for atherosclerotic cardiovascular disease (ASCVD) and calcific aortic valve stenosis. There are ongoing challenges with screening and management in primary and secondary prevention; however, future recommendations for clinical practice await the outcomes of clinical trials that are in progress.

## Introduction

**Figure 1.**
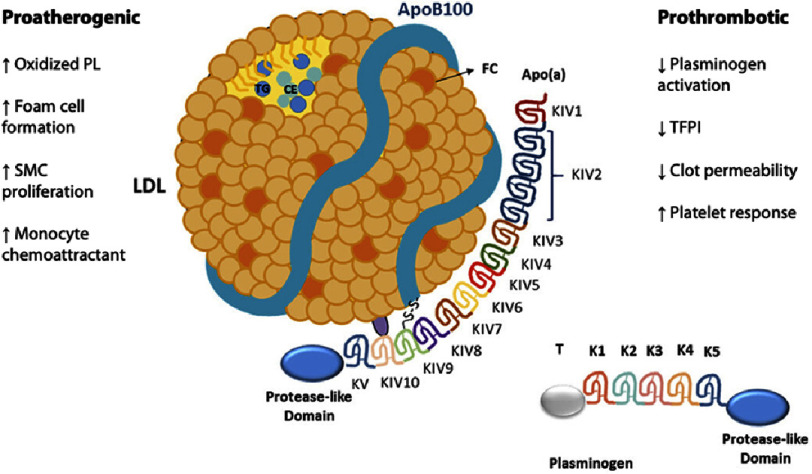
Structure, proatherogenic, and prothrombotic actions of lipoprotein(a). Adapted from https://doi.org/10.1002/mas.21747 under CC BY NC 4.0 licence.

Lp(a) was first described by Kare Berg in 1963^1^. It is a plasma lipoprotein comprising an ApoB100 molecule bound to glycoprotein(a) (Apo(a)) *via* a disulfide linkage (Figure 1). Apo(a) exhibits significant sequence homology with plasminogen however, unlike plasminogen it is not an active protease^2^. Apo(a) contains a high degree of variants in its polypeptide chain length due to the variable number of kringle domains^3^. An estimated 20–25% of the world’s population is believed to have elevated Lp(a) levels^4^. Lp(a) levels are genetically determined, and adult levels are typically reached by five years of age. Furthermore, Lp(a) levels are not modified by lifestyle factors such as exercise, dietary intervention, or weight optimization.

There is evidence that inflammatory conditions such as hypothyroidism, growth hormone therapy, kidney disease^8^ and pregnancy increase levels of Lp(a)^5–8^. Lp(a) levels decrease with hyperthyroidism, liver disease, and postmenopausal hormone replacement^9,10^.

Lp(a) is an established independent causal risk factor for ASCVD and calcific aortic valve stenosis supported by epidemiological studies, Mendelian randomization, and many prospective studies^11,12^. Lp(a) exhibits wide racial variations; individuals of African descent tend to have higher Lp(a) levels than those of white and Asian racial backgrounds^13^.

There is no generalized consensus on Lp(a) risk thresholds; however, it is apparent that the higher the Lp(a) concentration, the higher the cardiovascular risk. From a clinical perspective, these thresholds are useful and in demand. Levels below 75 nmol/L are considered normal, and above 125 nmol/L are considered raised in the European Atherosclerotic Society (EAS) consensus statement. A concentration above 100 nmol/L is accepted as a risk-enhancing cut-off in the National Lipid Association (NLA) scientific statement.

The main reason for screening patients for Lp(a) is to assist in the further identification of patients at high risk of cardiovascular disease. Currently, no specific Lp(a)-lowering medications have been approved for clinical use. The consensus recommendation among professional societies for Lp(a) management is appropriate control of other traditional risk factors. These include diabetes mellitus, hypertension, dyslipidemia, and changes in lifestyle (smoking cessation, increase in physical activity, healthier diet, and optimization of body weight). The implementation of these changes will contribute to a significant reduction in the global risk of ASCVD^20^.

The EAS recommends that everyone should have their Lp(a) tested at least once in their lifetime; however, screening has the potential to cause anxiety in patients found to have raised their Lp(a), particularly with the current limitations for specific interventions to address the problem. It is therefore vital that clinicians explain the indication for testing, the impact of raised Lp(a), and management strategies that can be employed before proceeding with testing. Screening in primary care may be beneficial for individuals with a significant family history of ASCVD to identify those who require more targeted lipid-lowering therapy initiated at a younger age.

Measuring Lp(a) is especially important in the context of secondary prevention for patients with recurrent cardiovascular events, where more aggressive lipid lowering may be indicated. Lipoprotein apheresis may be considered for patients with progressive ASCVD and raised Lp(a) levels, in whom regular apheresis has been demonstrated to result in a reduction in cardiovascular risk.

For decades, management of hypercholesterolemia has focused on low-density lipoprotein cholesterol (LDL-C). Multiple clinical trials of statin therapy and, more recently, bempedoic acid, ezetimibe, and PCSK9 Inhibitors have been shown to be effective in reducing LDL-C and ASCVD levels and improving outcomes. Currently, there are no approved Lp(a)-lowering medications. Statins have shown mixed results, and in some cases, they have been shown to increase Lp(a). No relevant reduction was observed with ezetimibe^14^.

Bempedoic acid slightly increased Lp(a) levels by 2.4%, but some studies found no effect on Lp(a) levels. Bile acid sequestrants and fibrates do not affect lipoprotein(a) levels. Niacin decreased Lp(a) levels by approximately 23%; however, its adverse effect profile and lack of outcome studies have limited its use, and it has not been approved in many countries.

PCKS9 inhibitors may be an option for lowering Lp(a). A meta-analysis showed that PCSK9 inhibitors lowered Lp(a) levels by 26% in addition to improving cardiac outcomes^15^. Lipoprotein apheresis treatment has been shown to be effective and beneficial in treating patients with elevated Lp(a) > 150 nmol/L (> 120 nmol/L in Germany) and progressive ASCVD, despite optimal treatment of all other risk factors. Treatment reduces Lp(a) levels by 60–80% per apheresis session; however, there is a rebound of Lp(a) levels that requires weekly or biweekly treatment. Many studies have concluded that regular apheresis has significant clinical benefits. However, with apheresis this not only reduces Lp(a) but also reduces LDL-C, other atherogenic lipoproteins and rheological parameters are improved^16,17,21^.

Unfortunately, awareness of the efficacy and benefit of lipoprotein apheresis is not widespread despite existing evidence of the success of apheresis treatment for raised Lp(a) levels, particularly in secondary prevention. Furthermore, it is simply unavailable in many countries.

### Novel Lp(a) lowering therapies in clinical trials

Specific Lp(a)-lowering therapies target the production of Apo(a) in liver cells using RNA-targeting strategies. The agents under investigation included single-strand antisense oligonucleotide (ASO) and short interfering RNA (siRNA). ‘Lp(a)Horizon’ is the first cardiovascular outcome study assessing the impact of Lp(a) lowering with pelacarsen on major cardiovascular events in patients with established cardiovascular disease. Pelacarsen reduces Lp(a) by 80% when taking 60–80 mg subcutaneously every four weeks^18^. It is estimated that this phase III cardiovascular outcome study will be completed by the end of 2026. The siRNA technology uses olpasiran, which reduces Lp(a) by approximately 95%^19^. The outcome study of olpasiran, ‘Ocean(a)-DOSE ’ is expected to be completed by the end of 2026. It will be interesting and intriguing to discover if specific significant Lp(a) lowering leads to improvement in cardiovascular outcomes, which will have a major impact on cardiovascular disease prevention.
